# Quality of life associated to chronic pelvic pain is independent of endometriosis diagnosis-a cross-sectional survey

**DOI:** 10.1186/1477-7525-9-41

**Published:** 2011-06-10

**Authors:** Carlos A Souza, Luciano M Oliveira, Camila Scheffel, Vanessa K Genro, Virginia Rosa, Marcia F Chaves, João S Cunha Filho

**Affiliations:** 1 Serviço de Ginecologia e Obstetrícia, Hospital de Clinicas de Porto Alegre, Porto Alegre, Brasil; 2 Serviço de Neurologia, Hospital de Clinicas de Porto Alegre, UFRGS, Porto Alegre, Brasil; 3 Departamento de Ginecologia e Obstetrícia, Universidade Federal do Rio Grande do Sul Porto Alegre, Brasil

**Keywords:** chronic pelvic pain, endometriosis, depression, anxiety, quality-of-life

## Abstract

**Background:**

Pain is strongly related to poor quality of life. We performed a cross-sectional study in a universitary hospital to investigate quality of life in women suffering from chronic pelvic pain (CPP) due to endometriosis and others conditions.

**Methods:**

Fifty-seven patients aged between 25 and 48 years-old submitted to laparoscopy because of CPP were evaluated for quality of life and depressive symptoms. Quality of life was accessed by a quality of life instrument [World Health Organization Quality of Life Assessment-Bref (WHOQOL-bref)]. Causes of pelvic pain were determined and severity of CPP was measured with a visual analogue scale. According to the intensity of pelvic pain score, patients were classified in two groups (group Low CPP < 25th percentile visual analogue scale and group High CPP > 25th percentile). Four dimensions on quality of life were measured (physical, psychological, social and environmental). We stratified the analysis of quality of life according CPP causes (presence or not of endometriosis in laparoscopy).

**Results:**

Patients with higher pain scores presented lower quality of life status in psychological and environmental dimensions. We found a negative correlation between pain scores and psychological dimension of quality of life (r = -0.310, P = .02). Quality of life scores were similar between groups with and without endometriosis (physical 54.2 ± 12.8 and 51.1 ± 13.8, P = 0.504; psychological 56.2 ± 14.4 and 62.8 ± 12.4, P = 0.182; social 55.6 ± 18.2 and 62.1 ± 19.1, P = 0.325; environmental 59.2 ± 11.7 61.2 ± 10.8, P = 0.608; respectively)

**Conclusions:**

Higher pain scores are correlated to lower quality of life; however the fact of having endometriosis in addition to CPP does not have an additional impact upon the quality of life.

## Background

Chronic pelvic pain (CPP) can be defined as a nonmalignant pain perceived in structures related to the pelvis; constant or recurring over a period of 6 months. In some cases it might be associated with negative cognitive, behavioral and social consequences [1]. The prevalence of CPP is variable according to the studied population; a populational study have demonstrated a rate of 3.8% [2], however in infertility samples this rate could be as high as 40% [3]. CPP is an important burden in women of reproductive age, with a direct impact on their marital, social and professional life [4,5]. Several papers with different methodological characteristics have shown an association of CPP with a negative impact on personal activities [6-8].

Endometriosis is a benign disease that mainly affects females during reproductive years and it is strongly associated with pelvic pain, being the most common gynaecological cause of CPP [9]. The strong association between endometriosis and pelvic pain was already demonstrated [10] and a discussion on nerve involvement as a factor contributing to pelvic pain in deep infiltrating endometriosis (DIE) has been published [9]. Although the physiopathology of pelvic pain in patients with endometriosis is still speculative, several clinical trials have documented that the treatment of endometriosis in these patients reduces associated symptoms [9,10]. Women with endometriosis presented more severe pain and greater social dysfunction than those with unexplained pain [11]. In addition, the proper treatment of endometriosis increases the quality of life and improves social behavior in this population [12]. Recently, authors have demonstrated that quality of life and sexual satisfaction of patients with CPP and endometriosis is altered [8].

Patients with CPP suffer from different pain intensities, and an important point is if patients with more intense pain have greater alteration in quality of life [7,13]. Others causes of CPP are also associated to alterations in perception of quality of life [13,14]. However, until this moment the complex relationship between the several causes of CPP and quality of life were not completely evaluated and questions remain unsolved. The aim of this study was to evaluate the influence of CPP on the quality of life and evaluate if endometriosis diagnosis *per se *adds a negative effect in patients' quality of life.

## Methods

### Design

We performed a cross-sectional study between April 2006 and December 2008.

### Patients

We included 66 consecutive patients complaining of pelvic pain aged between 25 and 48 years-old in the study. We defined pelvic pain as dysmenorrhoea and/or intermenstrual pelvic pain and/or dyspareunia of moderate to severe intensity lasting for more than 6 months [15]. We excluded patients with malignancies diagnosis (1), uterine myomas (2), ovarian cists (3), inflammatory pelvic disease (1), and pregnancy (2), so our final study population was 57 patients. The study was approved by the Ethical Committee of Hospital de Clínicas de Porto Alegre (IRB) and informed consent was obtained from all patients.

### Data collection

One month before laparoscopy, we collected baseline information (Age, gravity, vaginal labor, cesarean section, abortion and time of formal education), and patients answered an inventory about pelvic pain intensity. We interviewed all patients in order to evaluate: cognitive impairment, quality of life, anxiety symptoms, and depressive symptoms. After this initial evaluation, all patients were submitted to laparoscopy for pelvic pain investigation by the same investigator (JSLC). None of the patients had used oral contraceptives, progestagens, antinflamatory, antidepressives or psychotropic formulations in the three months that preceded the laparoscopy or Gonadotropin Releasing Hormone (GnRH) analogues in the last six months. Non-gynecologic causes of pelvic pain were excluded in all patients using history, physical examination and laboratory exams when appropriate.

### Instruments

The Visual Analogue Scale (VAS) was used to measure the mean pain intensity over the last three days (average for rest and activity). The 10-cm scale was marked with ''0'' (no pain) and ''10'' (worst possible pain), and the patients were instructed on how to use the scale [11]. Since CPP and endometriosis are two burden chronic diseases we chose to evaluate cognitive impairment in all patients. The exclusion of *c*ognitive impairment was done using the Mini Mental State Examination (MMSE) [16,17]. Quality of life was accessed by quality of life instrument [World Health Organization Quality of Life Assessment-Bref (WHOQOL-bref)], in an adequate translated and validated version [18]. It is a brief 26-item questionnaire, including 2 items for general quality of life and health status and another 24 items categorized into 4 domains (physical, psychological, social, and environmental). The item scores range from 1 to 5, with the higher score indicating the better quality of life on the corresponding item. The score for each domain ranges from 4 to 20, which is obtained by multiplying the average score of all items in this domain by the same factor of 4 [18]. Anxiety was evaluate by the *The Hamilton Anxiety Rating Scale (HARS)*[19]. The Brazilian version of the scale has showed to be reliable and valid, including 14 items on both physical and psychological symptoms [19]. Finally, depression was evaluated by the Beck Depression Inventory (BDI). The revised BDI is a 21-item self-assessment scale for eliciting severity of depression. Items score from 0 to 3. Reliability of internal consistency is good for mixed diagnoses as well as single and recurrent episode major depression [20]. The same investigator (LMO) applied all tests.

### Data analysis

According to pain score (VAS) we classified patients in two groups: High CPP (> 25^th ^VAS percentile) and Low CPP (< 25^th ^VAS percentile). We compared epidemiological, anxiety symptoms, depressive symptoms and quality of life (physical, psychological, social and environmental) between the groups. We also calculated the correlation between pelvic pain intensity and quality of life in the four dimensions. After this initial analysis, we classified patients according to the cause of pelvic pain. Patients were designated in two groups: endometriosis group and others' causes of pelvic pain group. The diagnosis of endometriosis was done using precise and standardized macroscopic criteria to make the visual diagnosis [21]. Endometriosis staging was carried out according to the classification of The American Society for Reproductive Medicine(ASRM) [22].

All statistics data were collected in a computerized database. Statistical analysis was performed using Statistical Package for the Social Sciences (SPSS) 13.0. The parametric data were presented as mean and mean standard deviation. Student's t test was carried out when appropriate. Pearson correlation was determined between pain scores and quality of life. *P-*value < .05 was considered statistically significant for all comparisons. The power of the study was calculated based on the following assumptions: (i) the sample size (n = 57), (ii) prevalence of endometriosis of 30% [23], (iii) expect difference of 8 points in each quality of life dimensions between the groups [24], (iv) type I and II errors of .05 and .19 respectively.

## Results

At the time of inclusion women were aged 35.8 ± 8.6 years and they showed average pain scores of 5.9 ± 2.9 on a 10 point scale.

### High CPP × Low CPP

Forty five patients presented pain scores consistent with the High CPP group (above the 75^th ^percentile) and 12 patients, with the Low CPP group (under the 25^th ^percentile). Age, duration of formal education, parity, number of vaginal deliveries and cesarean section remained similar irrespective of CPP groups (High and Low CPP). Unexpectedly, patients with High CPP demonstrated a lower number of abortions (1.2 ± 2.2 vs. 0.3 ± 0.7, P = .033)

We observed a statiscally significant reduction in quality life scores in patients classified in the High CPP group in the psychological (45.4 ± 15.6 *vs*. 58.2 ± 13.3, P = .007) and environmental (53.3 ± 10.7 *vs*. 60.7 ± 10.9, P = .044) domains. Scores in the psychological dimension and VAS were negatively correlated (r = -0.31, P = .02). However, we failed to demonstrate any correlation of other quality life domains and VAS (physical r = -0.078, P = .56; social r = -0.077, P = .573; and environmental r = -0.210, P = .104). The others variables measured-MMSE and BDI scores-were similar between the two groups.

### Endometriosis × other's pelvic pain

To clarify the role of endometriosis as a cause of CPP in quality of life scores patients were classified in two groups as stated by the cause of CPP diagnosed in laparoscopy: endometriosis group (study group), 32 patients; and others' causes of pelvic pain group (control group), 25 patients. All endometriosis were classified as minimal and mild endometriosis. These two groups were similar according epidemiological characteristics (Additional file 1 Table 1). Further, the MMSE, the BDI and the HARS scores were not affected by the cause of CPP. Moreover, scores from the VAS were similar between patients with endometriosis (6.7 ± 1.6) and those with others causes of CPP (7.5 ± 1.6) (Additional file 1 Table 2). Physical, psychological, social and environmental parameters of quality of life scale (WHOQOL-BREF) were similar between the groups.

## Discussion

In this study, we demonstrated that the intensity of pelvic pain is inversely correlated with alteration in the quality of life of patients with CPP (higher scores of pelvic pain were associated to decrease in quality of life in two domains-psychological and environmental). Furthermore, we showed that the presence of endometriosis as a cause of CPP is not an independent factor for modifying quality of life in patients with CPP.

Our study has some limitations: (i) all patients presented minimal/mild endometriosis according to ASRM criteria. Probably it was a selection bias because our service is a referral centre of infertility treatment and patients with more severe CPP or pelvic masses might have been directed to other services. Although endometriosis is an enigmatic disease and endometriosis classifications have poor correlation with clinical symptoms [25,26], patients with DIE or endometriomas could have presented more intense pain and differences in quality of life scores. This fact will remain to be elucidated since our sample does not contemplate these subjects. (ii) We compared patients with pelvic pain only, without the presence of a disease-free control group. This fact could have affected our results. Conversely, this approach has permitted to verify the importance of the intensity of pain in quality of life. The strength of our study lies in the fact that we evaluated patients under evaluation for CPP and use tools common to other forms of CPP and not only specific instruments for endometriosis. We decided not to use a specific instrument, described previously [7], to measure quality of life for patients with endometriosis because this would have introduced significant bias since our sample was also composed of patients without endometriosis [8]. Therefore, we have used an instrument that applies for all pelvic pain patients-the major resource of our investigation. Our results were, consequently, more comparable to other studies, and are in agreement with the results from authors that did not show any difference in terms of mood symptoms or personality characteristics when patients with endometriosis and unexplained pelvic pain were compared [11].

Authors showed that patients with chronic pelvic pain present some degree of hyperalgesia and some impairment of quality of life parameters (physical and mental) measured by SF-36 instrument [7]. Furthermore, these authors verified an increment in the intensity of pelvic pain in association with deterioration of quality of life. Unfortunately, we were not able to demonstrate differences in quality of life scores in all dimensions, only in psychological and environmental. Perhaps if we increase the number of patients in our groups we could be able to show this difference in all quality of life dimensions. Others, in a systematic review, demonstrated that mental health was the area of health-related quality of life that was most negatively affected by chronic pelvic pain whereas the least affected area was physical activities [6]. In addition, when pelvic pain is a primary symptom of gynecological diseases, like endometriosis it appears to have an even greater negative impact on health-related quality of life as compared to those conditions in which pelvic pain is not the most important symptom.

As a distinctiveness of our paper, we investigated the effect of endometriosis itself in quality of life of patients that presented with CPP. Pain is one of the major concerns of patients with endometriosis and its effect upon quality of life has been already shown [27] and we demonstrated that quality of life status was not affected by endometriosis *per se*. Infertility is another important characteristic of endometriotic patients. Although the number of pregnancies between the groups (endometriosis and without endometriosis) seems to be similar between the groups with, we can consider the possibility that infertility associated to endometriosis can also modify patients' quality of life. Authors have already demonstrated the diagnosis delay in endometriosis patients that suffer only from pain is almost twice the period of diagnosis for patients with infertility [25].

In accordance to our findings, a plethora of publications have shown that patients with chronic pelvic pain have a decreased quality of life and, consequently, reduced social adjustment with an increase in psychiatric morbidity [6,28], and recently alterations in sexual life [8]. Our data indicates that alterations in patient's quality of life are independent of the presence of endometriosis and they are in agreement with other authors that have demonstrated that CPP patients with or without endometriosis have no difference in results from quality of life and sexual life inventories [8]. The high prevalence of symptoms of anxiety and depression observed in both groups of this investigation was substancial. However, this finding was expected and consistent because our sample included only patients suffering from chronic pelvic pain. Moreover, it is important to emphasize that one of our objectives was to investigate the role of endometriosis in addition to pelvic pain as a determinant factor for quality of life [29,30].

## Conclusions

In conclusion, we showed that the cause of pelvic pain does not influence the quality of life status or anxiety-depression symptoms. Further, we demonstrated that pain intensity is correlated to a decrease in quality of life in psychological domain; and that patients with higher pelvic pain scores have lower quality of life in psychological and environmental dimensions. With this study we can speculate that management of pelvic pain of CPP patients, independent of the causal factor, is the best approach to improve quality of life of these patients.

## List of abbreviations

(ASRM): American Society for Reproductive Medicine; (BDI): Beck Depression Inventory; (CPP): Chronic Pelvic Pain; (DIE): Deep Infiltrating Endometriosis; (GnRH): Gonadotropin Releasing Hormone; (IRB): Institutional Review Board; (MMSE): Mini Mental State Examination; (SPSS): Statistical Package for the Social Sciences; (HARS): The Hamilton Anxiety Rating Scale; (VAS): Visual Analogue Score; (WHOQOL-bref): World Health Organization Quality of Life Assessment-Bref.

## Competing interests

The authors declare that they have no competing interests.

## Authors' contributions

CAS, LMO, JSL conceived and designed the study. CAS and MC analysed and interpreted the data. JSL supervised and reviewed all the statistical analysis. VR, VG, CS, MC contributed to data collection and performed surgical procedures. All the authors contributed to write the manuscript. All the authors approved the final version of the manuscript.
